# Reply to Šprajc: Sunrise alignments ensured farming success for the ancient inhabitants of the Basin of Mexico

**DOI:** 10.1073/pnas.2300250120

**Published:** 2023-04-03

**Authors:** Exequiel Ezcurra, Paula Ezcurra, Ben Meissner

**Affiliations:** ^a^Department of Botany and Plant Sciences, College of Natural and Agricultural Sciences, University of California Riverside, Riverside, CA 92521-0147; ^b^Climate Science Alliance, San Diego, CA 92169; ^c^Independent Filmmaker/Photographer, Sioux Falls, South Dakota 57104

We thank Šprajc (1) for his careful reading of our paper (2). He states, coinciding with us, that “the ancient inhabitants of the Basin of Mexico used prominent mountain peaks as markers of the Sun’s positions on agriculturally significant dates,” and finds that the astronomical and agricultural significance of our study is “hardly disputable.” His main point of criticism seems to be that, in his perspective, our study “resuscitates the unfounded idea that the Mexica employed a [calendric] intercalation system comparable to our leap years.” This is not our contention. On the contrary, we argue that by following solar alignment cues, the inhabitants of the Basin could have adjusted their calendar to the solar year without having to use an arithmetic leap-year algorithm.

We want to underscore two points in our paper: First, we restricted our study to the Basin of Mexico being aware that, as Šprajc suggests, the calendrical year was not necessarily synchronized throughout Mesoamerica. Second, we explored the question of calendric adjustments from an environmental and agricultural perspective, purposefully avoiding any hypothesis about ritual aspects. Some of the references that Šprajc argues that we should have cited were not included for these reasons, either because they deal with the larger Mesoamerica or the Maya region or because they concentrate on the 260-d ritual Mexica calendar and not on the solar calendar.

Šprajc contends that we subjectively selected only a few sources that support our views on calendric adjustments. Actually, we cited eight different sources with widely different views, from ancient chroniclers to Šprajc’s own work (3), to synthesize the leap-year debate. We tested our results against the chronology of Rafael Tena, the most cited modern author on this matter (4). The question we tried to address was how did the ancient people of the Basin of Mexico time the planting of their cornfields exactly at the end of an inhospitable dry spring and just before the beginning of a critically short rainy season. We hypothesized that they must have had an accurate farming calendar and noted that sunrise observed from Tepeyac, at the center of the Basin, aligns with Mount Tlaloc on February 23 to 24 and that the summit causeway of Mount Tlaloc aligns with sunrise on those same dates. These landmark alignments coincide with Tena’s date for the Basin’s new year. Our results agree with Šprajc’s assertion that “the orientations and alignments to horizon prominences enabled the use of easily manageable observational calendars, thus facilitating proper scheduling of agricultural […] activities.”

We agree with Šprajc that it seems unlikely that the Mexica could have adjusted their calendar using numerical intercalation like the Gregorian Calendar does. It seems more likely that they were using sunrise alignments to adjust their agricultural cycle to the solar seasons.

## References

[1] I. Šprajc, Astronomical alignments in the Basin of Mexico and a fixed correlation of the prehispanic calendar with the tropical year. Proc. Natl. Acad. Sci. U.S.A. **120**, e2221519120 (2023). 37011194 10.1073/pnas.2221519120 PMC10104581

[2] E. Ezcurra , Ancient inhabitants of the Basin of Mexico kept an accurate agricultural calendar using sunrise observatories and mountain alignments. Proc. Natl. Acad. Sci. U.S.A. **119**, e2215615119 (2022). 36508656 10.1073/pnas.2215615119 PMC9907100

[3] I. Šprajc, Problema del ajuste del año calendárico mesoamericano al año trópico. Anales de Antropología **34**, 133–160 (2000).

[4] R. Tena, El calendario mesoamericano. Arqueología Mexicana **41**, 4–11 (2000).

